# Correlative CD4 and CD8 T-cell immunodominance in humans and mice: Implications for preclinical testing

**DOI:** 10.1038/s41423-023-01083-0

**Published:** 2023-09-19

**Authors:** Tertuliano Alves Pereira Neto, John Sidney, Alba Grifoni, Alessandro Sette

**Affiliations:** 1 Center for Infectious Disease and Vaccine Research, La Jolla Institute for Immunology (LJI), La Jolla, CA 92037 USA; 2 Department of Medicine, Division of Infectious Diseases and Global Public Health, University of California, San Diego, La Jolla, CA 92037 USA

**Keywords:** T cells, SARS-CoV-2, Vaccine, Animal testing, HLA, Cellular immunity, Vaccines

## Abstract

Antigen-specific T-cell recognition is restricted by Major Histocompatibility Complex (MHC) molecules, and differences between CD4 and CD8 immunogenicity in humans and animal species used in preclinical vaccine testing are yet to be fully understood. In this study, we addressed this matter by analyzing experimentally identified epitopes based on published data curated in the Immune Epitopes DataBase (IEDB) database. We first analyzed SARS-CoV-2 spike (S) and nucleoprotein (N), which are two common targets of the immune response and well studied in both human and mouse systems. We observed a weak but statistically significant correlation between human and H-2^b^ mouse T-cell responses (CD8 S specific (*r* = 0.206, *p* = 1.37 × 10^−13^); CD4 S specific (*r* = 0.118, *p* = 2.63 × 10^−5^) and N specific (*r* = 0.179, *p* = 2.55 × 10^−4^)). Due to intrinsic differences in MHC molecules across species, we also investigated the association between the immunodominance of common Human Leukocyte Antigen (HLA) alleles for which HLA transgenic mice are available, namely, A*02:01, B*07:02, DRB1*01:01, and DRB1*04:01, and found higher significant correlations for both CD8 and CD4 (maximum *r* = 0.702, *p* = 1.36 × 10^−31^ and *r* = 0.594, *p* = 3.04^−122^, respectively). Our results further indicated that some regions are commonly immunogenic between humans and mice (either H-2^b^ or HLA transgenic) but that others are human specific. Finally, we noted a significant correlation between CD8 and CD4 S- (*r* = 0.258, *p* = 7.33 × 10^21^) and N-specific (*r* = 0.369, *p* = 2.43 × 10^14^) responses, suggesting that discrete protein subregions can be simultaneously recognized by T cells. These findings were confirmed in other viral systems, providing general guidance for the use of murine models to test T-cell immunogenicity of viral antigens destined for human use.

## Introduction

Several lines of evidence have demonstrated that T cells contribute to the modulation of disease severity in the context of SARS-CoV-2 infection and associated diseases [1–3] and, in certain cases, can also contribute to preventing (re)infection [4–6]. Similar observations have been made for several other acute viral infections, including influenza viruses [7–11] and poxviruses [12–15]. Accordingly, the inclusion of antigens designed to induce T-cell responses, in parallel and with synergism with antibody responses, can be considered to optimize vaccine efficacy and durability [16]. This might be accomplished by immunogens, such as whole antigens, protein domains, subfragments, or discrete epitopes, selected for optimal T-cell activity in humans [16]. However, the development of such vaccine constructs requires preclinical testing for immunogenicity in relevant animal models, for which murine models are commonly used. In this context, it is important to address the extent to which optimizing T-cell activity in animal models would reflect optimal T-cell immunogenicity in humans, and, conversely, whether constructs with optimal T-cell activity in humans would be sufficiently immunogenic in animal models to allow for meaningful preclinical testing.

T-cell immunogenicity depends on a complex series of variables [17–21]. Prominent roles are played by the level of expression of different antigens that can potentially be recognized, the kinetics and location of expression during the infectious cycle [22–24], access to endogenous and exogenous processing pathways, the relative susceptibility of different protein domains to cellular processing, and the capacity of different epitope sequences to bind major histocompatibility complex (MHC) proteins, allowing presentation for T-cell scrutiny. Apart from MHC binding, as discussed in the next paragraph, most of these variables can be reasonably expected to be similar in humans and mice, providing an intrinsic rationale for murine testing of immunogens intended for human use. In fact, studies have shown that in the case of complex antigens of some viruses, such as poxviruses and herpes viruses, antigens that are frequently recognized in humans also tend to be recognized in murine systems [17, 25, 26].

MHC binding capacity is necessary but not sufficient for T-cell immunogenicity [27, 28]. The genes encoding MHC molecules are polygenic and polymorphic and are known as Human Leukocyte Antigen (HLA) in humans and the H-2 complex in mice [29, 30]. In humans, the HLA complex encodes four different main types of class II heterodimers (DRB1, DRB3/4/5, DP, and DQ) and three main distinct types of class I molecules (A, B, and C). Each of these seven different molecules is highly polymorphic, and each allelic variant presents distinct sets of peptide epitopes.

The overall organization of the murine H-2 complex is similar, but not identical, to that of the human HLA complex. For example, the H-2 complex does not encode a DP ortholog, and certain strains of mice lack the L class I locus (ortholog of HLA-C in humans) or do not express functional class II IE molecules (orthologs of HLA-DR) [31, 32]. Although MHC polymorphisms evolved after the separation of murine and human MHC [33, 34], little overlap has been reported between the epitopes presented by different allelic variants of HLA and the murine H-2 complex, though some examples of overlap have been reported in certain cases due to convergent evolution [33]. Therefore, it cannot be expected priori that epitopes or regions that are immunogenic in humans would also be recognized in murine models, potentially posing a challenge to preclinical testing and optimization of T-cell components of vaccines.

Nevertheless, it has been reported that certain regions within a given protein antigen have the capacity to bind several different MHC types [35–37]. These “hot spots” might originate from a particular structural feature of the antigen in question and/or from the presence of sequences capable of binding multiple MHC types with overlapping peptide binding capacities [38–40]. It was indeed shown that immunodominant regions are associated with “promiscuous binding”, as defined as the capacity to bind multiple MHC types [41–43]. Hence, it would be reasonable to hypothesize that immunodominant regions that are frequently recognized in humans in the context of different HLAs might also have the capacity to bind MHC molecules expressed in common murine strains. If this is the case, testing human immunodominant antigenic regions in murine systems might be feasible.

A popular alternative is the use of HLA transgenic mice [44–46], with a T-cell repertoire showing overlapping recognition with what is expressed in humans [47–49]. However, this approach has limitations, namely, apparent competition of endogenous murine MHC, suboptimal interaction of HLA molecules with murine class I and class II [47], and availability for only a limited number of HLA allelic variants. Additionally, HLA-transgenic mice generally express only one HLA molecule, which does not provide a stringent replica of the diversity of epitopes presented for recognition by human T cells.

In the present study, we analyzed the patterns of HLA class I and class II immunogenicity related to most studied SARS-CoV-2 spike (S) and nucleoprotein (N) antigens, as well as other viral antigens, in humans and murine systems. The results will directly address and guide planning for murine testing of viral T-cell antigens destined for human use.

## Materials and methods

### T-cell epitope data retrieval strategy

The analysis was based on the immunogenicity data curated in the Immune Epitope DataBase (IEDB) (www.iedb.org) [50]. For our analysis, we focused on spike (S) and nucleoprotein (N) antigens. These are two of the antigens of SARS-CoV-2 most frequently targeted by the immune system and widely studied [24], thus providing good overall antigen coverage in terms of epitope identification and characterization. We queried the database on 02/23/2023. As search criteria, we specified the organism SARS-CoV-2 (organism ID: 2697049) and the antigen Spike glycoprotein (Uniprot ID: P0DTC2) or Nucleoprotein (Uniprot ID: P0DTC9). Our analysis involved T-cell assays, with both positive (actual epitopes) and negative (peptides tested but negative) data included while excluding B-cell assays and MHC binding or elution assays [51]. For each antigen of interest, we established query parameters related to two specific hosts: mice (*Mus musculus*) and humans (*Homo sapiens*). To explore each immunogenicity pattern, we concentrated our analysis on queries of murine H-2^b^ class I and class II molecules and the global set of human HLA class I or HLA class II molecules. Within the human subset, we placed a particular emphasis on responses restricted by the HLA alleles A*02:01 and B*07:02 for class I and DRB1*01:01 and DRB1*04:01 for class II, as these alleles are prevalently utilized in HLA transgenic mice. In addition to specific human HLA-restricted data, we included data, when available, from transgenic mice corresponding to the alleles listed above. Finally, to investigate the potential correlation in immunodominance patterns of epitopes restricted by both murine H-2^b^ haplotypes and human HLA alleles across different viruses, we expanded our analysis to include proteins from non-SARS-CoV-2 viruses, as detailed in Table 1. Our search considered proteins with a minimum of 50 epitopes identified as of 03/03/2023. These query sets covered the two class I alleles in humans (HLA-A*02:01 and B*07:02) and the murine H-2^b^ class I haplotype. Similarly, the class II human alleles (DRB1*01:01 and DRB1*04:01) and mice H-2^b^ class II were included in the analysis. Overall, the resulting epitopes derived from the queries of each additional protein were compared with the global human HLA class I or II epitope list.

**Table 1:** Non-SARS-CoV-2 viruses and proteins investigated

Organism (ID)	Protein (UniProt ID)
Dengue Virus (12637)	Polyprotein (P17763)
Epstein Barr Virus (10376)	Nuclear Antigen 1 (P03211)
Hepatitis C Virus (11103)	Polyprotein (P27958)
Influenza A Virus (11320)	Polymerase Acid Protein (P03433)
Influenza A Virus (11320)	Nucleoprotein (P03466)
Influenza A Virus (11320)	Polymerase Basic Protein 2 (P03428)
Influenza A Virus (11320)	Hemagglutinin (P03452)
Influenza A Virus (11320)	RNA Polymerase Catalytic Subunit (P03431)
West Nile Virus (11082)	Polyprotein (Q9Q6P4)
Yellow Fever Virus (11089)	Polyprotein (P03314)
Zika Virus (64320)	Polyprotein (Q32ZE1)

Evaluation of immunodominance pattern correlations of epitopes was performed across murine and human MHC alleles

### Response frequency acquisition and data analysis

The ImmunomeBrowser tool, which is freely available at IEDB Analysis Resource (IEDB-AR) [51], was used to extract and process information on T-cell immunogenicity in mice (H-2^b^) and humans. This tool calculates the response frequency (RF) for each residue along the sequence of interest, indicating the frequency with which a specific residue has been associated with positive responses in a target protein position. In essence, the tool maps queries of results from IEDB and combines the frequency of responses from different subjects, tests, and reports from the curated scientific literature. However, RF values can only be calculated for residues for which data (positive or negative) are available, and no RF values are available for residues without associated data. RF values were obtained for each of the described queries. By overlaying the RF scores from different datasets, we were able to visualize the extent of overlap in immunogenicity patterns between humans and mice or between the HLA restrictions and the global human HLA data. Additionally, to quantify the level of similarity in immunogenic profiles across different models or MHC restrictions, we calculated the percentage of shared residues that exhibited an RF greater than or equal to 0.1 in both queries. This approach enabled us to measure the proportion of residues that matched in both datasets of the total protein sequence while meeting the RF threshold.

### Statistical analysis

To evaluate the correlation strength between humans and mice, as well as HLA restrictions and the global human HLA class I or II, we performed Spearman rank correlation analyses on the RF values obtained for each dataset with a 95% confidence interval using GraphPad Prism version 9.5.1. Statistical significance was determined by *p* value below 0.05, and scientific annotation was used to visualize *p* values. In our analysis of non-SARS-CoV-2 proteins, we compared the resulting *r* coefficient for each protein in specific restriction or cross-species datasets to the corresponding global human HLA data. Additionally, we assessed differences in the distribution of these coefficients by the unpaired nonparametric Mann‒Whitney test (*p* < 0.05) using GraphPad 9.5.1.

## Results

### Correlation of immunogenicity patterns of SARS-CoV-2 S and N class I-restricted epitopes in humans and H-2^b^ mice

To determine whether the immunodominance patterns of epitopes restricted by murine and human MHC class I correlate, on 02/23/2023, we extracted data curated in IEDB [50] pertaining to SARS-CoV-2 S- and N-derived epitopes. We similarly queried for epitopes tested in mice and restricted by class I molecules expressed in the H-2^b^ haplotype, which is characteristic of commonly utilized mouse strains, such as C57BL/6. Regarding S, 960 distinct epitopes were tested for human HLA class I and 199 for H-2^b^ mice. For N, 211 epitopes were tested in humans and 59 in mice. The lower amount of data associated with the H-2^b^ murine system was not surprising because T-cell responses to S and N have been studied extensively in humans. Next, we utilized the ImmunomeBrowser tool [51] to map epitopes to reference protein sequences.

**Fig. 1:**
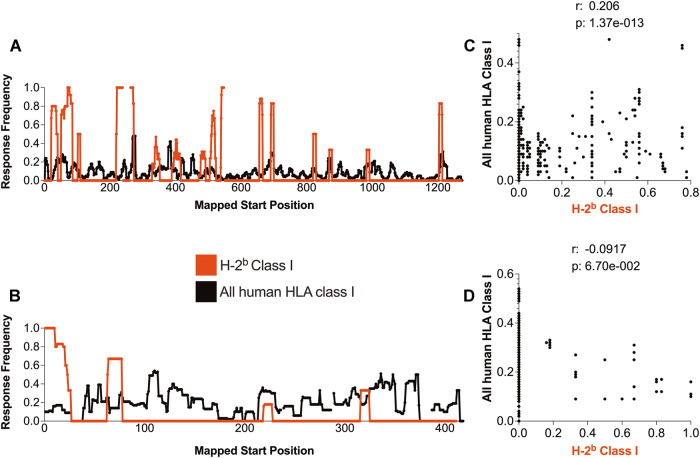
SARS-CoV-2 S and N antigen immunodominance patterns for class I restricted epitopes in humans and H-2^b^ mice. The response frequency of murine (H-2^b^; red) and human (black) class I-restricted epitopes for S (**A**) and N (**B**) SARS-CoV-2 antigens were calculated using the ImmunomeBrowser tool available at IEDB. Spearman’s correlations between mouse and human data are also shown for S (**C**) and N (**D**)

We conducted comparative analysis of the data from mice and humans to identify regions of the proteins that showed reactivity in both systems. To assess the density of the reactivity of sequences across the protein, we evaluated the RF values obtained with the ImmunomeBrowser tool, which aggregates epitope data within a reference protein. Class I epitope data exported from the ImmunomeBrowser are plotted in Fig. 1A, B. Our results showed that mice can recognize many of the same epitopes as humans but that the specificity is not always identical. To assess the degree of similarity in terms of immunogenicity, across species or MHC restrictions, we also calculated the percentage of common sequences between two queries. In this analysis, we focused on sequences that exhibited an RF greater than 0.1 in both systems. This approach allowed us to quantify the percentage of matching residues, and specifically, we found that 9.97% of the residues in the S antigen and 4.08% in the N antigen are associated with recognition in both humans and mice. Certain regions, such as S51-68 and N316-324, are reported to be immunogenic in both human and murine systems. However, other regions, such as S700-819 and N105-121, have been reported to be immunogenic in humans but not in mice; conversely, S477-489 is recognized in mice but not in humans. Spearman correlation analysis indicated a moderate (R = 0.206), but highly significant, correlation for S (*p* = 1.37e-013), though no correlation was noted for *N* (R = −0.092, *p* = 6.70e-002) (Fig. 1C, D). These results should be interpreted with the caveat that, as more data become available, certain regions not currently reported as immunogenic might indeed be found to be immunogenic in humans or H-2^b^ mice.

### Correlation of immunogenicity patterns of S and N class II-restricted epitopes in humans and H-2^b^ mice

**Fig. 2:**
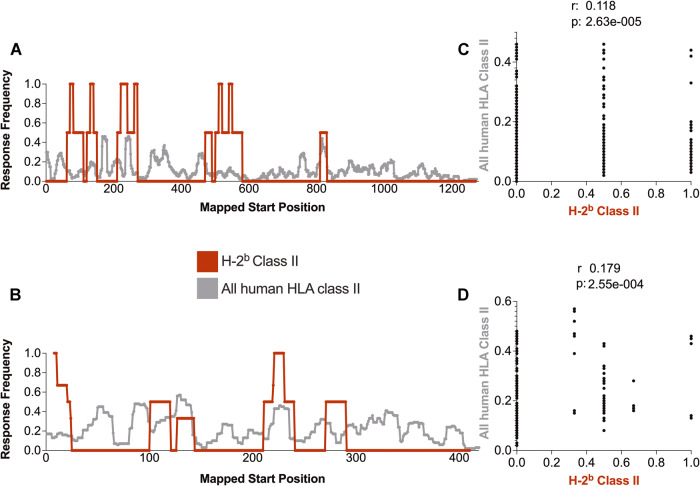
SARS-CoV-2 S and N antigen immunodominance patterns for class II restricted epitopes in humans and H-2^b^ mice. The response frequency of murine (H-2^b^; maroon) and human (gray) class II-restricted epitopes for S (**A**) and N (**B**) SARS-CoV-2 antigens were calculated using the ImmunomeBrowser tool available at IEDB. Spearman’s correlations between mouse and human data are also shown for S (**C**) and N (**D**)

Next, we examined the potential correlation between the human and murine immunogenicity patterns for HLA and H-2^b^ class II. As of 02/23/2023, data were available for a total of 941 distinct S-derived epitopes tested in human HLA class II and 124 in H-2^b^ class II. For N, 193 distinct epitopes have been similarly tested in humans and 42 in mice. The class II epitope data exported from the Immunobrowser tool are plotted in Fig. 2A, B.

The results showed that certain regions, such as S221-240 and N101-120, are immunogenic in both human and murine systems. Other regions, such as S831-944, have been reported to be immunogenic in humans but not in H-2^b^ mice. Spearman correlation analyses (Fig. 2C, D) indicated a significant correlation between humans and H-2^b^ mice for both S (*p* = 2.63e-005) and N (*p* = 2.55e-004), with *r* values of 0.118 (S) and 0.179 (N). Additionally, the percentage of shared epitopes accounted for 10.53% of the S and 7.78% of the N antigen, supporting the weak level of correlation.

### Correlation of immunogenicity patterns of S and N general class I reactivity with HLA-A*02:01 and HLA-B*07:02 restricted epitopes

**Fig. 3:**
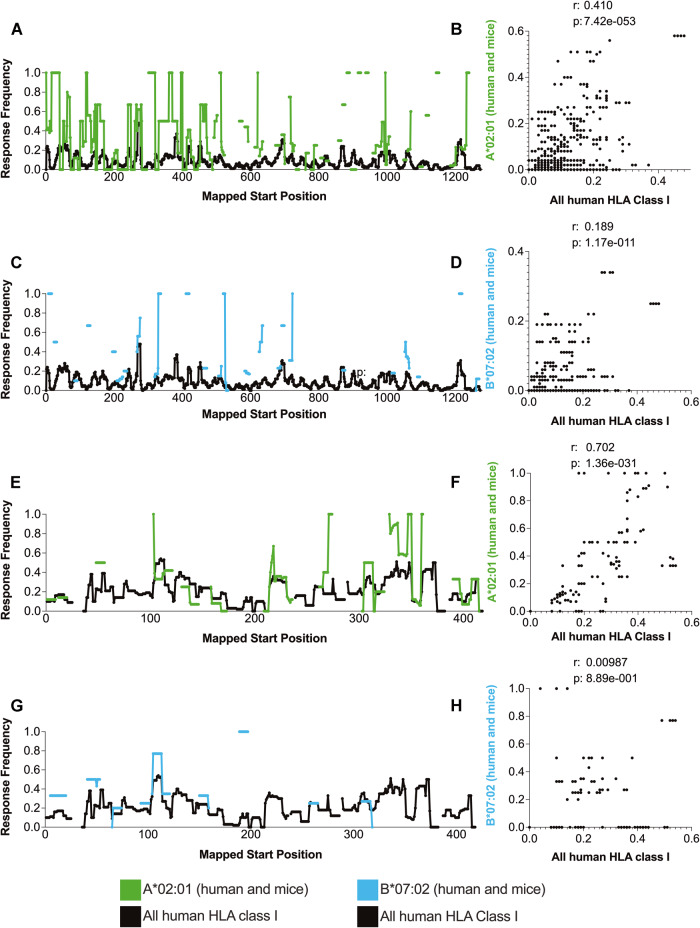
Comparative dominance of HLA A*02:01 and HLA B*07:02 epitopes over general HLA class I in SARS-CoV-2 S and N antigens. The response frequency of human nonspecific HLA class I (black) and transgenic mouse HLA class I A*02:01 (green) and B*07:02 (turquoise) epitopes for S (**A**, **B**) and N (**C**, **D**) SARS-CoV-2 antigens were calculated using the ImmunomeBrowser tool available at IEDB. Spearman’s correlations between mouse and human data are also shown for S (**E**, **G**) and N (**F**, **H**)

Two HLA class I molecules, A*02:01 and B*07:02, have been frequently studied in human populations and are commonly used in HLA transgenic mouse testing [52, 53]. Accordingly, we extracted epitopes restricted to A*02:01 and B*07:02 from any host, which might include data from both human and HLA transgenic mouse studies. These extracted epitopes were compared with the overall HLA class I epitopes derived exclusively from human assays. For the S antigen, we found 153 epitopes for A*02:01 and 43 for B*07:02 and compared these data to the full set of 960 epitopes for HLA class I by mapping the response frequency (RF) per amino acid residue across the corresponding sequence regions. The correlations observed were highly significant (*p* = 7.42e-053 and *p* = 1.17e-011, with *r* values of 0.410 and 0.189 for A*02:01 and B*07:02, respectively), as depicted in Fig. 3A–D, suggesting that these two HLA restrictions are reliable representatives of global human HLA class I. Furthermore, we found that 22.94% of the residues restricted to A*02:01 and 9.58% to B7*07:02 epitopes overlap with the global human HLA data simultaneously.

Additionally, we analyzed the correlation between data only from transgenic mouse HLA A*02:01 and exclusively from human HLA A*02:01, with significance (*p* = 2.13e-03, *r* = 0.174) (Fig. S1A). However, we did not find any data on the S antigen for transgenic mouse B*07:02. This finding supports the use of transgenic mice in studying HLA A*02:01-restricted human epitopes, especially considering the large amount of data available for this HLA allele in IEDB.

With regard to the N antigen, we found 76 A*02:01- and 19 B*07:02-restricted epitopes in queries including human and transgenic mouse data and a total of 211 for all human HLA class I data. Of the total A*02:01-restricted epitopes, 12.18% were also found to be reactive in the overall human HLA data. Similarly, 8.01% of the B*07:02-restricted epitopes identified in humans and transgenic mice were also reactive in the global data. Significant correlations were detected for A*02:01 (*p* = 1.36e-031, *r* = 0.702) but not for B*07:02 (*p* = 8.89e-01; *r* = 0.009), as illustrated in Fig. 3E–H. We also analyzed the correlation between transgenic mice and human A*02:01 data separately but observed only four epitopes restricted to transgenic mice. Thus, we can infer that the limited data for mice specific for the N antigen contribute to the lack of correlation between the datasets (*p* = 8.89e-001, *r* = −0.322) (Fig. S1B). Additionally, we did not find any epitopes restricted to B*07:02 in the query targeting only transgenic mice. Furthermore, our data indicate similarities but also relevant differences in immunodominance patterns between the HLA class I alleles and global human data. These results emphasize that careful consideration of the specific region and protein being investigated is necessary to determine the most suitable transgenic mice to use for experiments.

### Correlation of immunogenicity patterns of S and N general class II reactivity with HLA DRB1*01:01 and DRB1*04:01 restricted epitopes

As mentioned above, HLA transgenic mice provide a valuable animal model for testing HLA-restricted epitopes with activity detected in human subjects. Here, we explore the extent to which the epitope repertoire of two of the most common HLA class II alleles for which HLA transgenic mice are available (HLA DRB1*01:01 and DRB1*04:01) [54–56] mirrors the CD4 reactivity of the general human population reported in the literature and curated in IEDB.

**Fig. 4:**
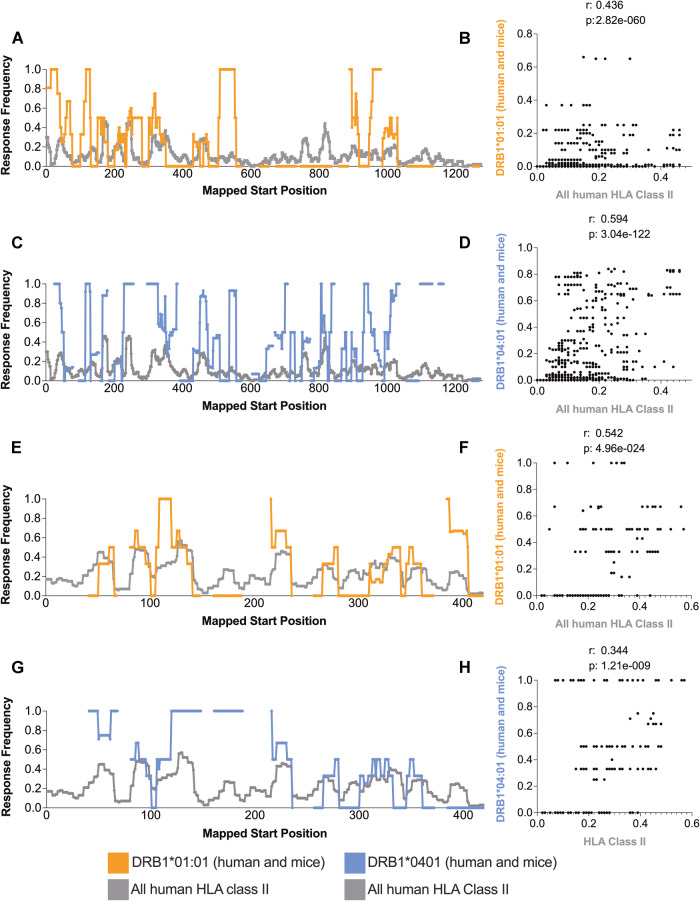
Relative dominance of HLA DRB1*01:01 and HLA DRB1*04:01 epitopes versus general HLA class II in SARS-CoV-2 S and N antigens. The response frequency of human nonspecific HLA class II (gray) and transgenic mouse HLA class II DRB1*01:01 (orange) and DRB1*04:01 (purple) epitopes for S (**A**, **B**) and N (**C**, **D**) SARS-CoV-2 antigens were calculated using the ImmunomeBrowser tool available at IEDB. Spearman’s correlations between mouse and human data are also shown for S (**E**, **G**) and N (**F**, **H**)

The data related to the S antigen for DRB1*01:01 and DRB1*04:01, including human and transgenic mouse data, accounted for 114 and 134 epitopes, respectively, and a total of 941 epitopes for all class II human data curated in IEDB. Among the epitopes, we found 25.29% and 35.04% of the DRB1*01:01 and DRB1*04:01 restricted residues, respectively, to be immunogenic in both queries. We also observed significant correlations between DRB1*01:01 and DRB1*04:01 compared to the total human HLA class II epitopes (*p* = 2.82e-060 and *p* = 3.04e-122 and *r* values of 0.436 and 0.594 for DRB1*01:01 and DRB1*04:01, respectively) (Fig. 4A–D). Moreover, we observed a significant (*p* = 8.97e-007, *r* = 0.264) (Fig. S1C) correlation between the separate data for transgenic mice and humans restricted to HLA DRB1*01:01; no correlation was observed for DRB1*04:01 restriction (*p* = 4.99e-001, *r* = −0.0396) (Fig. S1D).

Similar analyses were conducted for *N*, with 26 and 39 restricted epitopes for DRB1*01:01 and DRB1*04:01, respectively. We compared the numbers to the total number of 182 epitopes restricted for all class II human data curated in IEDB and found significant correlations (*p* = 4.96e-024 and *p* = 1.21e-009) and *r* values (*r* = 0.542 and *r* = 0.344) for DRB1*01:01 and DRB1*04:01, respectively (Fig. E–H). Specifically, 13.20% and 15.08% of the residue reactivity associated with DRB1*01:01 and DRB1*04:01, respectively, are shared between the two HLA queries of the N antigen. However, it is important to note that no data exclusively from transgenic mice were available for both class II alleles analyzed for the *N* antigen. Taken together, these results show that HLA-DR variants display a certain degree of similarity in patterns of reactivity. Although transgenic mice are valuable models for studying the human immune response, the appropriate strain depends on the region of the protein being analyzed.

### Correlation between HLA class I and class II reactivity in human datasets for S and N

An additional consideration in the design of T-cell-inducing vaccines is whether the same antigen or antigenic region(s) can effectively induce both CD8 and CD4 responses. In the case of SARS-CoV-2, both S and N antigens are major targets for both responses [57, 58]. However, it should not be assumed that the same antigens are dominant, as exemplified by the case of pox viruses and flaviviruses, for which the antigens most dominant for CD8 and CD4 only partially overlap [59, 60]. Furthermore, as cellular processing pathways are largely distinct between CD4 and CD8 epitopes, it is possible that the CD4 and CD8 immunodominant regions within a protein antigen might be largely distinct.

**Fig. 5:**
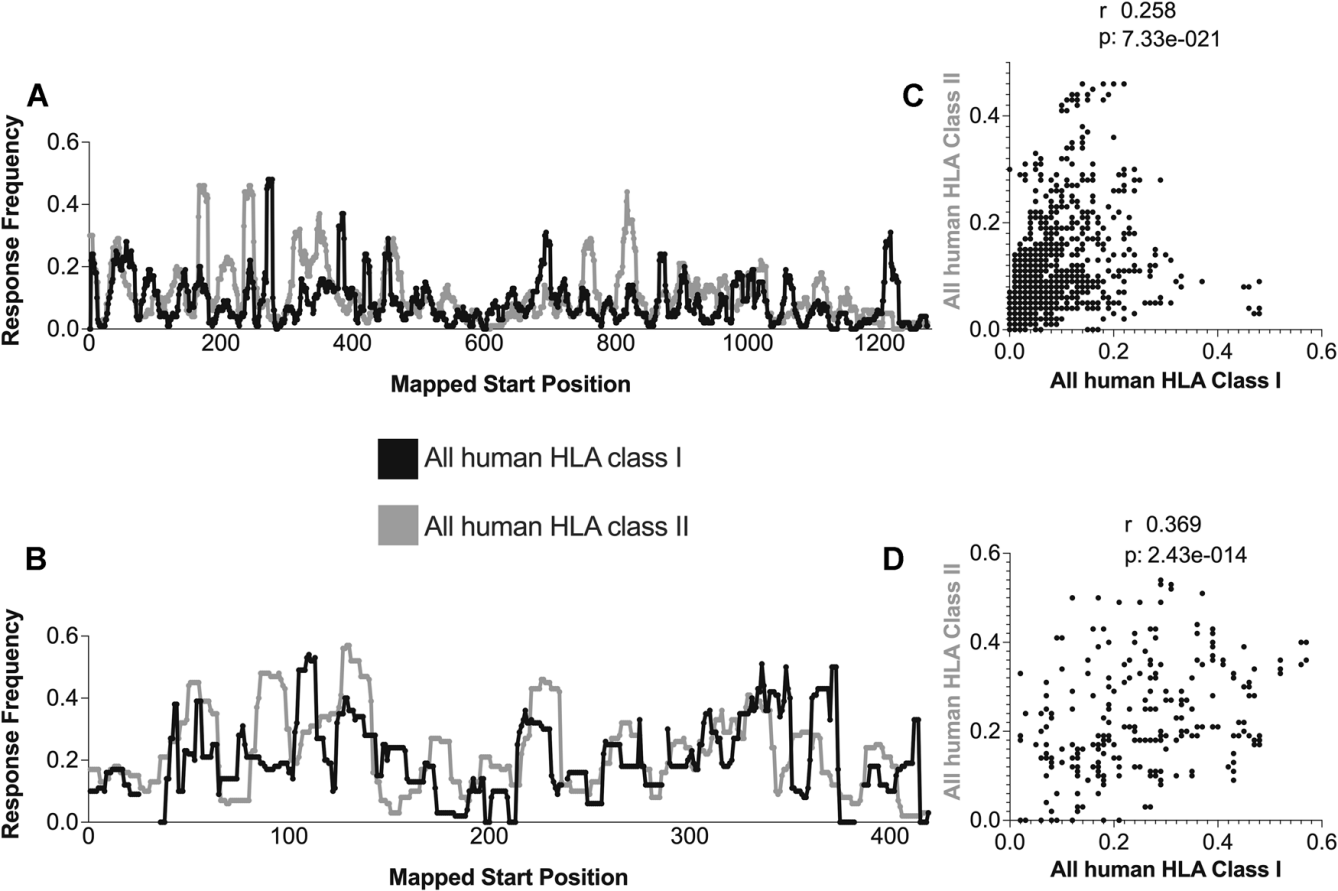
Correlation between reactivity patterns of class I and class II epitopes in human HLA data SARS-CoV-2 S and N antigens. The response frequency of human nonspecific HLA class I (black) and class II (gray) for S (**A**) and N (**B**) SARS-CoV-2 antigens were calculated using the ImmunomeBrowser tool available at IEDB. Spearman’s correlations between mouse and human data are also shown for S (**C**) and N (**D**)

To address this issue, we overlaid HLA class I and class II reactivity plots for S and N (Fig. 5A–B). Surprisingly, the results identified several regions immunogenic for both class I and class II restrictions, and these accounted for 20.82% of the S and 23.10% of the N sequences. For instance, the 341-378 region of the S antigen was found to be immunogenic for both CD4 and CD8 T cells. Spearman correlation values were highly significant (Fig. 5C, D) for S and N (*p* = 7.33e-021 and *p* = 2.43e-014, respectively), with *r* values in the range of 0.258 to 0.369. These results suggest that protein regions with overlapping reactivity might be targeted to simultaneously induce both CD4 and CD8 responses.

### Correlation of immunodominance in human and murine class I and II epitopes across non-SARS-CoV-2 viruses

Next, we investigated whether the immunodominance patterns of epitopes restricted by both murine and human MHC alleles correlate with other non-SARS-CoV-2 viruses. To this end, we employed a data-driven approach by querying IEDB data for virus antigens with more than 50 epitopes, including positive and negative assays restricted by MHC class I or class II. This resulted in the selection of 11 MHC class I and six MHC class II systems for further analysis.

**Fig. 6:**
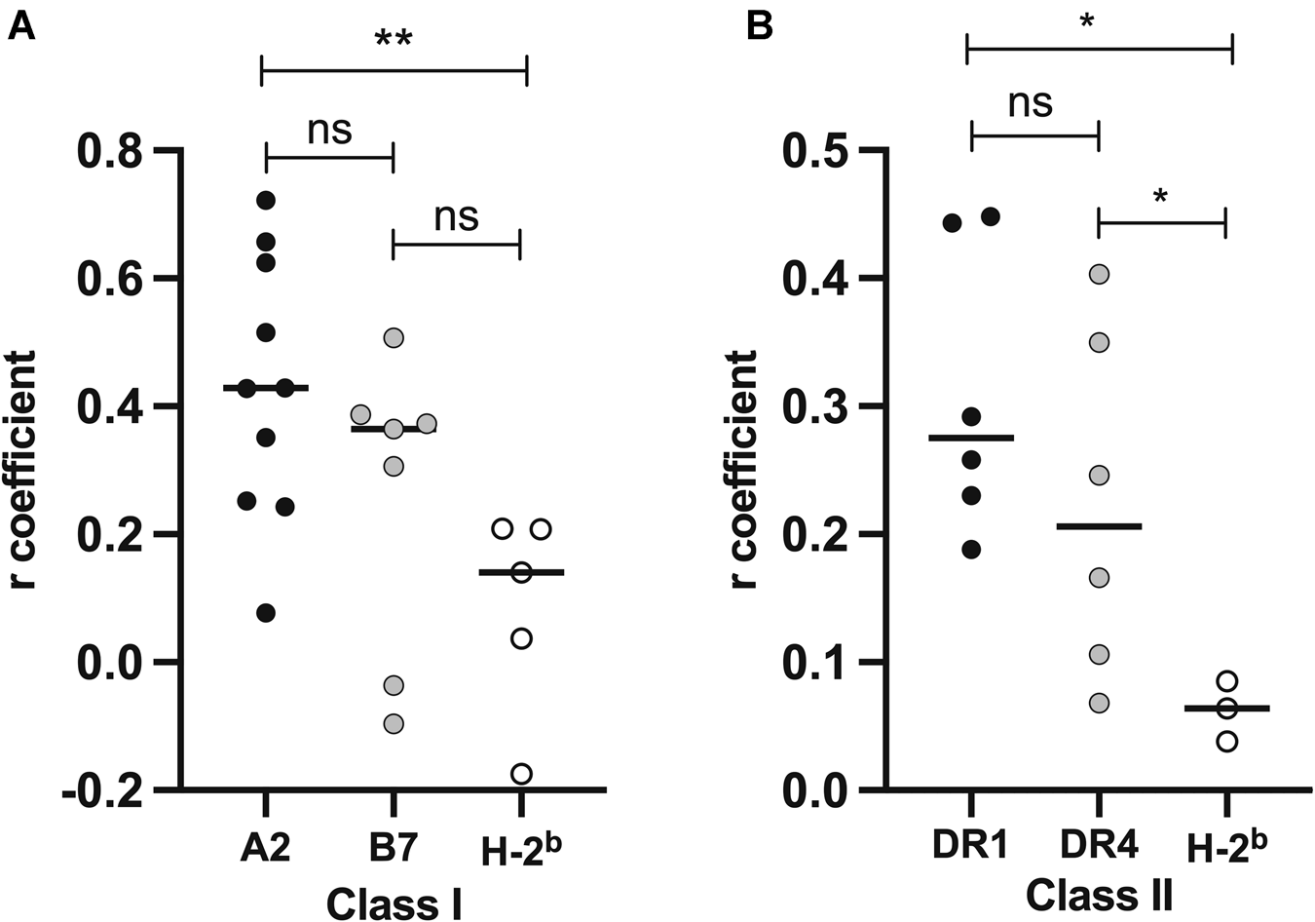
Differences in correlation coefficient (R) as a function of class I and class II restrictions. The R coefficient for each protein was obtained by comparing selected class I and class II human alleles or murine H2^b^ haplotypes to the global human HLA of the corresponding class which is shown in Table 1. Differences in the distribution of values among the different groups were assessed using the Mann‒Whitney test. Each dot corresponds to the correlation coefficient of one viral antigen. Black dots: (**A**) A*02:01 (**B**) DRB1*01:01, gray dots: (**A**) B*07:02 (**B**) DRB1*04:01, white dots: (**A**, **B**) H-2^b^ mouse haplotypes. *p < 0.05; **p < 0.01; ns not significant

For each of the viral species selected, we then compared the immunodominance patterns of A*02:01, B*07:02, and class I H-2^b^ epitopes with the global human HLA class I reactivity. Similarly, DRB1*01:01, DRB1*04:01, and class II H-2^b^ epitopes were compared with global HLA class II. Our analysis revealed a significant correlation between most antigens and global human HLA, though with a weak to moderate strength (Table 2). Interestingly, we observed that the overall correlation coefficient for the different antigens in each restriction query was slightly higher for A*02:01 and DRB1*01:01 (0.077 to 0.722 and 0.188 to 0.448, respectively). Further investigation revealed that the A*02:01, DRB1*01:01, and DRB1*04:01 alleles had significantly higher *r* values than the murine H-2^b^ strain (*p* = 5.00e-03, *p* = 2.00e-02, and *p* = 5.00e-02, respectively) (Fig. 6A, B). These findings suggest that utilizing the A*02:01, DRB1*01:01, and DRB1*04:01 alleles may provide a more accurate and improved representation of the overall T-cell response in humans and might be prioritized in animal testing for vaccine design.

**Table 2:** Correlation of epitopes derived from multiple viral antigens

	A*02:01		B*07:02		H-2^b^ Class I	
Virus (Protein)	*r*	*p* value	*r*	*p* value	*r*	*p* value
Influenza A Virus (Nucleoprotein)	0.429	7.64E-19	0.373	2.54E-10	0.209	5.63E-04
Influenza A Virus (Polymerase basic protein 2)	0.243	7.64E-10			0.208	5.18E-03
Dengue Virus (Polyprotein)	0.351	9.06E-33	0.096	1.42E-03		
Hepatitis C Virus (Polyprotein)	0.657	2.21E-204			0.14	2.24E-04
West Nile Virus (Polyprotein)	0.077	5.78E-02	0.036	3.76E-01		
Yellow Fever Virus (Polyprotein)	0.625	1.69E-65	0.507	2.83E-38		
Epstein Barr Virus (Nuclear antigen 1)	0.515	5.31E-09	0.387	1.40E-07		
Influenza A Virus (Polymerase acid protein)	0.428	8.75E-09				
Influenza A Virus (RNA polymerase catalytic subunit)	0.252	3.40E-05	0.306	4.78E-06		
Influenza A Virus (Hemagglutinin)	0.722	1.90E-55			0.175	6.45E-03
Zika Virus (Polyprotein)			0.365	5.25E-13	0.037	2.76E-01

	DRB1*01:01		DRB1*04:01		H-2^b^ Class II	
	*r*	*p* value	*r*	*p* value	*R*	*p* value
Dengue Virus (Polyprotein)	0.443	8.87E-53	0.106	1.94E-06	0.064	2.94E-02
Influenza A Virus (Hemagglutinin)	0.188	5.20E-06	0.35	3.24E-18	0.038	3.67E-01
Zika Virus (Polyprotein)	0.292	1.94E-49	0.246	3.20E-35	0.085	5.14E-07
Hepatitis C Virus (Polyprotein)	0.448	5.07E-25	0.403	3.00E-19		
Influenza A Virus (Nucleoprotein)	0.23	2.08E-07	0.166	1.65E-03		
Yellow Fever Virus (Polyprotein)	0.258	2.62E-13	0.068	2.68E-02		

The analysis was based on epitopes derived from viral antigens with more than 50 records in IEDB and focused on A*02:01, B*07:02, and H2^b^ class I or DRB1*01:01, DRB1*04:01 and H2^b^ class II restrictions. The Spearman R coefficient and *p* values for each antigen were obtained by comparing the specific allele or haplotype to the global human HLA of the corresponding class

## Discussion

The basis for exploring an inclusive T-cell vaccine approach is supported by evidence that highlights the role of T-cell responses in controlling viral infections and diseases, such as COVID-19, and reducing disease severity [1, 2]. Furthermore, T-cell responses observed across viral variants [61, 62] indicate potential for targeting epitopes that broaden the range of protective immune responses. Once such epitopes are identified, preclinical testing in animals, typically mice or nonhuman primates, is essential to assess the safety and efficacy of vaccine constructs and their capacity to elicit an immune response that justifies further testing in humans [63]. Although animal models are crucial for understanding the immune response, their ability to accurately predict vaccine effectiveness in humans is often limited [64]. Therefore, aligning the selected vaccine system with a more discerning choice of species or strains for animal models should enhance translation of the response observed in mice to humans.

In light of this, the current study provides additional insights into the association between human and murine T-cell responses. Our data show that the targets of human and murine H-2^b^ class I- and class II-restricted CD4 and CD8 T-cell responses correlate weakly but significantly. We also found that the epitope targets recognized in the overall human population correlate with those restricted by common HLA types for which HLA transgenic mice are available. This suggests that common HLA alleles are a good model for the overall immune response and that HLA transgenic mice can be used to study reactivity to a particular antigen in a setting that more closely mimics the human response. We also showed that, perhaps unexpectedly, the same regions tend to be recognized by CD4 and CD8 responses. These results were first observed for SARS-CoV-2 S and N antigens but were subsequently confirmed in other antigenic systems, including influenza virus, dengue virus, yellow fever virus, Zika virus, West Nile virus, Epstein‒Barr virus, and hepatitis C virus.

Finding a correlation between human and murine T-cell targets was not expected a priori, given that the MHC binding specificity of murine and human MHC molecules is generally distinct [31, 32]. This can be explained by previous observations reporting that certain antigens frequently recognized in humans are also frequently recognized in mice with diverse haplotypes, suggesting that other factors, such as cellular processing or structural features of the antigens recognized, underlie this effect [65]. Although convergent evolution is also a possibility [33, 34], it is rare when considering humans and mice.

In our analysis, we showed that certain regions are recognized in both humans and H-2^b^ mice, indicating that vaccine constructs destined for human use and incorporating these regions can be tested in this murine haplotype. A limitation of our study was that only the immunogenicity of H-2^b^ mice was considered. This is because insufficient data were available in IEDB for other mouse strains, or even nonhuman primates (NHPs), to allow for meaningful analysis. Similar analyses can be performed for other mouse haplotypes or other animal species as more data become available. Therefore, in light of these limitations, our results may not be generalizable to other mouse strains or nonhuman primates. Nonetheless, the fact that we found regions that are recognized in both humans and H-2b mice is encouraging, as it suggests that these regions may be important for eliciting protective immunity in different species.

We also show that the epitope targets recognized in the overall human population correlate with those restricted by common HLA types for which HLA transgenic mice are available. HLA A*02:01 and DRB1*01:01 are highly representative of the global human HLA response [66–68], indicating their suitability for testing human vaccine constructs. An important caveat in interpreting these results is that epitopes restricted by these common alleles are overrepresented in the data available in the scientific literature and curated by IEDB [50]. However, our approach ensured an unbiased analysis by covering all the IEDB data available without prioritizing any allelic variants. Based on the published literature, and thereby reflecting historical trends, a significant portion of IEDB-curated data generally pertains to alleles predominant in Caucasian populations. Presently, and as previously noted [69], the A*02:01 A*24:02, B*07:02, A*01:01, A*11:01, and A*03:01 alleles are the most frequently identified restriction elements for class I epitopes derived from SARS-CoV-2 proteins. These alleles are all frequent in Caucasian populations, and they include the two (or more) most frequent alleles in North African, Northeast Asian, Southeast Asian, Southwest Asian, and Oceanian populations, as well as the top or second most frequent allele in Sub-Saharan African, South American, and Aboriginal populations. Indeed, analysis of the number of SARS-CoV-2 epitopes with defined HLA restriction revealed that, contrary to the past, the SARS-CoV-2 data do not appear to be biased toward identification of epitopes restricted by alleles specifically prevalent in Caucasians but instead correlate highly with allelic frequencies in the overall worldwide population [70]. Similarly, most of the class II restrictions found are for alleles common in Caucasians, such as DRB1*04:01, DRB1*15:01, DRB1*01:01, DRB1*11:01, and DRB1*07:01. In this case, these alleles are common in most worldwide population groups, and epitope restriction data analysis also suggested a higher correlation with allelic frequencies in the overall worldwide population. Regarding HLA transgenic mice, however, the strains for which most of the experimental data are available represent allelic variants primarily prevalent in Caucasians, except for A*02:01. HLA transgenic mice representative of additional class I and II alleles [71, 72] have been developed, yet limited data from these additional strains have been reported thus far.

It is notable, however, that some of the alleles included in our analysis are prototype representatives of HLA-supertype specificities, reflecting allelic clusters frequent in the global population that share overlapping epitope binding repertoires. Considering supertype relationships, it is possible that the epitope sets identified to date still reflect a broader population representation. For instance, A*02:01, the most prevalent allele worldwide, including in the majority of non-Western populations, belongs to the HLA-A2 supertype, with frequencies ranging from 39 to 45.9% in different populations [68, 73]. HLA-B*07:02 is also relatively frequent in most populations, including African (7.3%) and Asian (4.4%) populations [41], and is representative of the HLA-B7 supertype, globally found with frequencies in the 43–57% range [68]. Similarly, for class II, HLA DRB1*01:01 is mainly observed in Caucasians, and it is associated with a diverse repertoire [39]. Binding to DRB1*01:01 has been utilized to efficiently identify T-cell epitopes restricted across a diverse set of class II alleles [74–76]. We previously reported significant bias in that most data available derive from a limited set of HLA alleles [77]. This bias in epitope identification studies was also recently noted in the context of alleles frequent in indigenous populations [78]. It is desirable that this bias be corrected; as more studies define epitopes restricted by other alleles, it will be possible to extend and repeat the present analyses to include additional alleles, particularly those represented in a diverse set of ethnic backgrounds [39, 79].

Nevertheless, our data demonstrate that use of HLA transgenic mice allows for closer mimicking of the overall pattern of the antigens and antigen regions recognized by human T-cell responses compared to that observed for the H-2b murine system.

We observed significant correlations between the CD8 and CD4 T-cell antigen regions recognized. This was somewhat unexpected, given that rather different processing pathways preside over the generation of HLA class II- and class I-restricted epitopes [80]. Nevertheless, there are several reports of “hot spot” regions [35–38, 40] broadly recognized by class I/CD8- and class II/CD4-restricted T cells. Such shared antigen regions indicate the potential efficacy of a peptide-based vaccine containing both CD8 and CD4 epitopes. However, small peptide vaccines may provide only a limited MHC restriction profile, which may in turn limit their recognition by the immune system in both individuals and the broader population while potentially exhibiting suboptimal immunogenicity and reduced efficacy due to inadequate antigen presentation by presenting cells [81]. These limitations can be overcome by vaccine constructs that include epitopes able to induce both responses or by use of longer peptides that contain multiple additional CD8 and CD4 epitopes [82, 83].

Finally, the fact that the basic observations made in the case of the S and N SARS-CoV-2 antigens were also noted in additional viral antigens indicates their general applicability. Overall, our findings demonstrate that detectable and significant correlations exist between CD4 and CD8 responses restricted by different MHCs. This suggests that vaccines and immunogens based on selected subregions destined for human use can be tested in murine models, though these correlations should not be taken for granted, and animal testing of a particular human immunogenic region requires judicious selection of a suitable animal model system. This type of analysis is, for example, relevant in the context of potential development of vaccine constructs encompassing regions conserved across different viral families of pandemic concern and immunogenic in humans for preclinical testing in suitable animal models.

### Supplementary information

Correlation analyses between data from transgenic mice and human

Supplementary Figure 1
