# Correction: Correlative CD4 and CD8 T-cell immunodominance in humans and mice: implications for preclinical testing

**DOI:** 10.1038/s41423-023-01099-6

**Published:** 2023-11-14

**Authors:** Tertuliano Alves Pereira Neto, John Sidney, Alba Grifoni, Alessandro Sette

**Affiliations:** 1 Center for Infectious Disease and Vaccine Research, La Jolla Institute for Immunology (LJI), La Jolla, CA 92037 USA; 2 Department of Medicine, Division of Infectious Diseases and Global Public Health, University of California, San Diego, La Jolla, CA 92037 USA

**Keywords:** Cellular immunity, Vaccines

Correction to: *Cellular & Molecular Immunology* 10.1038/s41423-023-01083-0, published online 19 September 2023

The article “Correlative CD4 and CD8 T-cell immunodominance in humans and mice: Implications for preclinical testing”, written by Tertuliano Alves Pereira Neto, John Sidney, Alba Grifoni & Alessandro Sette, was originally published electronically on the publisher’s internet portal on 19 September 2023 without open access. With the author(s)’ decision to opt for Open Choice the copyright of the article changed on 26 October 2023 to © The Authors 2023 and the article is forthwith distributed under a Creative Commons Attribution 4.0 International License, which permits use, sharing, adaptation, distribution and reproduction in any medium or format, as long as you give appropriate credit to the original author(s) and the source, provide a link to the Creative Commons licence, and indicate if changes were made. The images or other third party material in this article are included in the article’s Creative Commons licence, unless indicated otherwise in a credit line to the material. If material is not included in the article’s Creative Commons licence and your intended use is not permitted by statutory regulation or exceeds the permitted use, you will need to obtain permission directly from the copyright holder. To view a copy of this licence, visit http://creativecommons.org/licenses/by/4.0.

