# Supplementary figures and images for: Correlative CD4 and CD8 T-cell immunodominance in humans and mice: Implications for preclinical testing
Source: Cell Mol Immunol. 2023 Sep 19;20(11):1328–38. doi: 10.1038/s41423-023-01083-0 (PMC10616275; doi:10.1038/s41423-023-01083-0)

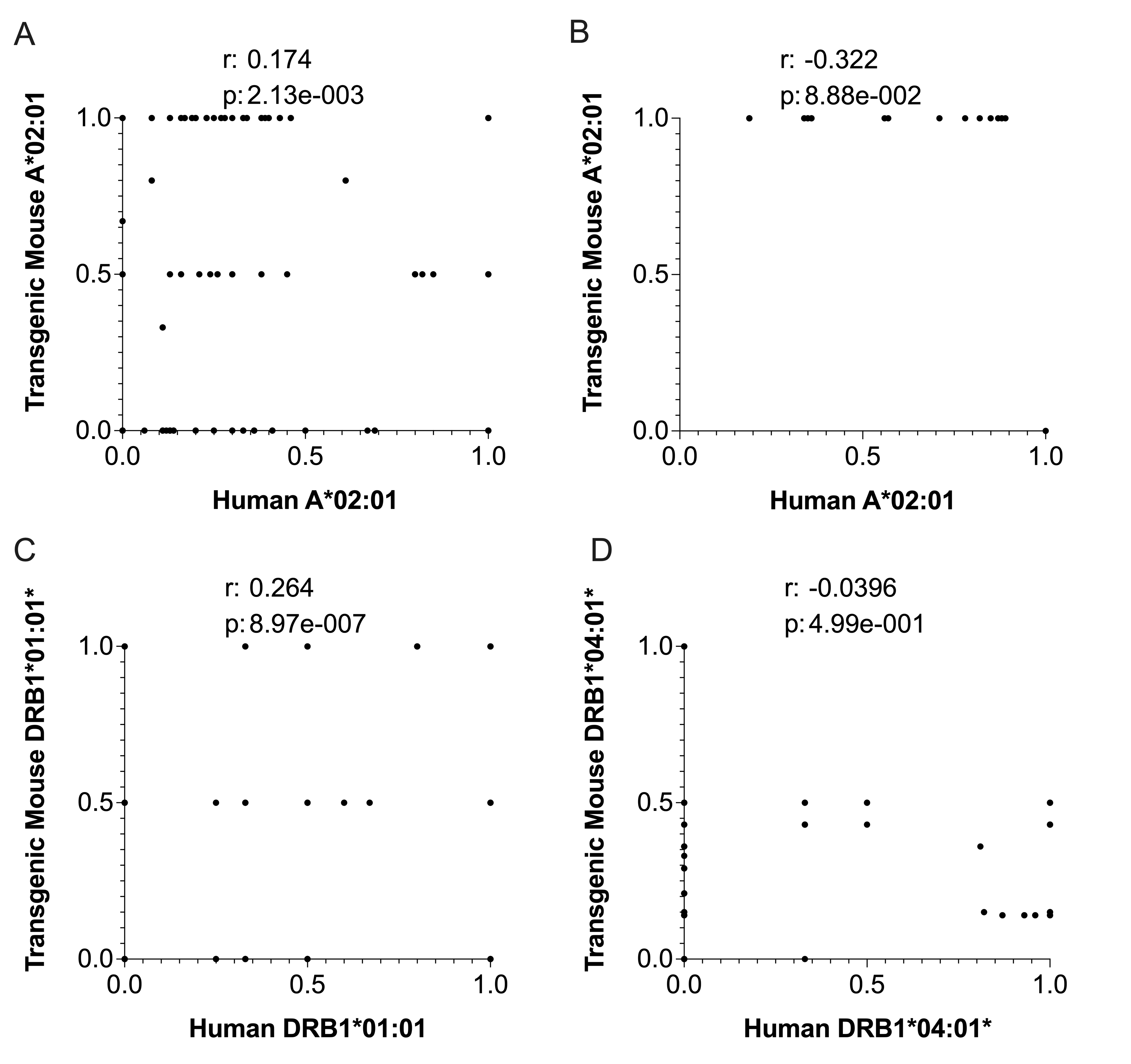

Supplement: Supplementary file 1 — Correlation analyses between data from transgenic mice and human [file 41423_2023_1083_MOESM1_ESM.tif]
