# Supplementary material for: Correlative CD4 and CD8 T-cell immunodominance in humans and mice: Implications for preclinical testing
Source: Cell Mol Immunol. 2023 Sep 19;20(11):1328–38. doi: 10.1038/s41423-023-01083-0 (PMC10616275; doi:10.1038/s41423-023-01083-0)
Supplement: Supplementary file 2 — Supplementary Figure 1 [file 41423_2023_1083_MOESM2_ESM.docx]

**Supplementary Figure 1. Correlation analyses between data from transgenic mice and humans.** Class I A*02:01 (upper panels) S (A) and N (B) epitopes and class II (lower panels) DRB1*01:01 (C) and DRB1*04:01 (D) restricted S epitopes were compared between mouse and human data to evaluate the correlation level using Spearman's rank correlation analysis with a 95% confidence interval.
